# Pattern‐triggered immunity restricts host colonization by endophytic fusaria, but does not affect endophyte‐mediated resistance

**DOI:** 10.1111/mpp.13018

**Published:** 2020-11-18

**Authors:** Francisco J. de Lamo, Margarita Šimkovicová, David H. Fresno, Tamara de Groot, Nico Tintor, Martijn Rep, Frank L. W. Takken

**Affiliations:** ^1^ Molecular Plant Pathology Faculty of Science Swammerdam Institute for Life Sciences University of Amsterdam Amsterdam Netherlands

**Keywords:** *Arabidopsis*, Avr2, endophyte‐mediated resistance, *Fusarium oxysporum*, tomato, wilt disease

## Abstract

*Fusarium oxysporum* (Fo) is best known as a host‐specific vascular pathogen causing major crop losses. Most Fo strains, however, are root endophytes potentially conferring endophyte‐mediated resistance (EMR). EMR is a mechanistically poorly understood root‐specific induced resistance response induced by endophytic or nonhost pathogenic Fo strains. Like other types of induced immunity, such as systemic acquired resistance or induced systemic resistance, EMR has been proposed to rely on the activation of the pattern‐triggered immunity (PTI) system of the plant. PTI is activated upon recognition of conserved microbe‐associated molecular patterns (MAMPs) of invading microbes. Here, we investigated the role of PTI in controlling host colonization by Fo endophytes and their ability to induce EMR to the tomato pathogen Fo f. sp. *lycopersici* (Fol). Transgenic tomato and *Arabidopsis* plants expressing the Fo effector gene *Avr2* are hypersusceptible to bacterial and fungal infection. Here we show that these plants are PTI‐compromised and are nonresponsive to bacterial‐ (flg22) and fungal‐ (chitosan) MAMPs. We challenged the PTI‐compromised tomato mutants with the EMR‐conferring Fo endophyte Fo47, the nonhost pathogen Fom (a melon pathogen), and with Fol. Compared to wild‐type plants, *Avr2*‐tomato plants became hypercolonized by Fo47 and Fom. Surprisingly, however, EMR towards Fol, induced by either Fo47 or Fom, was unaffected in these plants. These data show that EMR‐based disease resistance is independent from the conventional defence pathways triggered by PTI, but that PTI is involved in restricting host colonization by nonpathogenic Fo isolates.

## Graphical abstract

Whereas MAMP‐triggered immunity restricts growth of pathogenic and endophytic *Fusarium oxysporum*, it does not restrict the endophyte‐mediated resistance conferred by the latter.

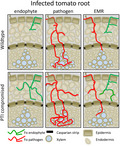

## 1 INTRODUCTION

Vascular wilt pathogens cause large losses in important agronomical crops (Michielse & Rep, 2009; Yadeta & Thomma, 2013). The vascular wilt pathogen *Fusarium oxysporum* (Fo) has a wide host range of over 100 crops, yet each *forma specialis* (f. sp.) infects one or a few related plant species only (Edel‐Hermann & Lecomte, 2019; Michielse & Rep, 2009). The main symptoms of fusarium wilt disease are vascular browning, leaf epinasty, and wilting (Agrios, 2005). Fo enters roots by hyphal swellings through wounds, lateral roots emergence points, or the root tip depending on the Fo strain and its host. Thereafter, hyphae grow mainly apoplastically through the root cortex eventually reaching the vasculature from which the fungus colonizes the aboveground tissues (Gordon, 2017). Control of wilt diseases is difficult as no curative treatments are available once the plant is infected. In addition, chlamydospores, being the major resting structures of the fungus, can remain viable in the soil for decades. To date, the best strategy to control wilt disease is the use of resistant crops. Unfortunately, genetic resistance is not available in many plant species (Yadeta & Thomma, 2013).

Plants are constantly challenged by potentially pathogenic microbes and the roots in particular are exposed to a high diversity of microorganisms (Hacquard et al., 2017). To counteract microbes, plants recognize microbe‐associated molecular patterns (MAMPs) through pattern recognition receptors (PRRs) located at the cell surface (Macho & Zipfel, 2014). Recognition of MAMPs starts a broad‐spectrum immune response called pattern‐triggered immunity (PTI) (Bigeard et al., 2015; Jones & Dangl, 2006). PTI induces early signalling responses, like Ca^2+^ and H^+^ influx, production of reactive oxygen species (ROS), and phosphorylation of mitogen‐associated protein kinases (MAPKs), as well as late responses such as callose deposition (Bigeard et al., 2015; Chuberre et al., 2018). To counteract PTI and to promote host colonization, microbes secrete effector proteins. Some of these effectors are detected by plant resistance (R) proteins, resulting in the induction of effector‐triggered immunity (ETI) (Jones & Dangl, 2006). R proteins can be of different classes. Most of them are intracellular nucleotide‐binding leucine‐rich repeat (NLR)‐type receptors (NLRs), but receptor‐like proteins or kinases (RLP and RLK, respectively) have also been identified that specifically recognize effectors (Catanzariti et al., 2017; Fradin et al., 2009; Postma et al., 2016). The tomato (*Solanum lycopersicum*) *R* genes *I*, *I‐2*, *I‐3*, and *I‐7* that confer resistance to Fol encode an RLP, an NLR, and two RLKs, respectively (Catanzariti et al., 2015, 2017; Gonzalez‐Cendales et al., 2016; Simons et al., 1998). These receptors mediate recognition of the effectors Avr1 (Six4), Avr2 (Six3), and Avr3 (Six1) from the tomato‐infecting Fo f. sp. *lycopersici* (Fol) (Houterman et al., 2008). Some of these resistances have already been broken in the field by the emergence of new races: Fol race 2 isolates have overcome *I*‐mediated resistance by deletion of *Avr1*, and race 3 isolates that have overcome *I‐2*‐mediated resistance contain an *Avr2* gene with either a point mutation or a small deletion (Biju et al., 2017; Houterman et al., 2009; Takken & Rep, 2010).

Besides genetic resistance in the host, biocontrol is an alternative method to control wilt diseases. Many studies have shown that endophytic Fo strains can confer protection to pathogenic Fo isolates (de Lamo & Takken, 2020). A potential advantage of this method is that protection does not rely on single *R* genes and hence might be more durable and more difficult to overcome by the pathogen. A caveat of biocontrol is that the level of resistance is typically not as high as that conferred by resistance genes (de Lamo et al., 2018). Fo‐based biocontrol is the sum of different components. The first component consists of the endophyte directly mycoparasitizing or competing with the pathogen for nutrients and root entry points, limiting progression of the latter. Another component encompasses a root‐specific immune response triggered by Fo endophytes (de Lamo & Takken, 2020), and we refer to this response as endophyte‐mediated resistance (EMR).

In tomato, EMR is induced independent of the defence hormones jasmonic acid (JA), ethylene (ET), and salicylic acid (SA) (Constantin et al., 2019). The colonization pattern of wild‐type and hormone mutants by the Fo47 endophyte is identical and also EMR is unaffected in tomato plants impaired in SA accumulation (*NahG*), JA biosynthesis (*def1*), or ET‐production (*ACD*) and ‐sensing (*Nr*) (Constantin et al., 2019). The independence of EMR on SA, JA, and ET implies that EMR might be distinct from the known induced immune responses that do rely on either SA (systemic acquired resistance, SAR) or JA/ET (induced systemic resistance, ISR) (Fu & Dong, 2013; Pieterse et al., 2014). These latter responses are preceded by a PTI response, and therefore we set out to investigate whether PTI is involved in EMR and/or colonization of the host by Fo.

To study the link between EMR and PTI we used tomato as model. As a PTI tomato mutant we employed a transgenic line that expresses the Fol effector gene *Avr2*. *Avr2*‐expressing plants show compromised PTI responses such as growth inhibition, ROS production, and callose deposition upon treatment with the bacterial MAMP‐derivative flg22 (Di et al., 2017). Lines expressing *Avr2* are also hypersusceptible to bacterial and fungal pathogens, suggesting that besides bacterial MAMP‐induced PTI the plant responses to fungal MAMPs are also compromised. Here, we describe that in *Avr2* plants PTI triggered by the fungal MAMP chitosan is compromised as well.

As an established model system for EMR, the tripartite interaction between tomato, Fo47, and Fol, was used (Aïcha et al., 2014; Aimé et al., 2013; Bolwerk et al., 2005; Constantin et al., 2019; de Lamo et al., 2018). Besides in tomato, Fo47 confers resistance to Fo pathogens in various plant species, such as asparagus (*Asparagus officinalis*; Elmer, 2004), flax (*Linum usitatissimum*; Trouvelot et al., 2002), and eucalyptus (*Eucalyptus viminalis*; Salerno et al., 2000), and to the vascular pathogen *Verticillium dahliae* and root‐infecting oomycetes *Phytophthora capsici* in pepper (*Capsicum annuum*; Veloso & Díaz, 2012) and *Pythium ultimum* in cucumber (Benhamou et al., 2002). Unlike Fol, Fo47 is a poor root‐ and stem colonizer and stem colonization is typically restricted to the crown and although it effectively colonizes roots the amount of fungal biomass is approximately 20‐fold lower than that of a pathogenic strain (Constantin et al., 2020). Notably, whereas stem and root colonization by Fol are reduced by about 9‐fold upon coinoculation with Fo47, the endophyte becomes a better stem colonizer in the presence of the pathogen despite not colonizing the roots more extensively (Constantin et al., 2020). Besides Fo47, which has a purely endophytic lifestyle, we also included in our bioassays the nonhost pathogen Fo f. sp. *melonis* (Fom), which typically infects melon. Together these experiments allowed us to investigate the role of PTI in EMR, and in colonization of tomato by the endophyte Fo47 and by Fom.

## 2 RESULTS

### 2.1 Endophyte‐mediated resistance can be conferred by endophytic and nonhost isolates of *F. oxysporum*

**FIGURE 1:**
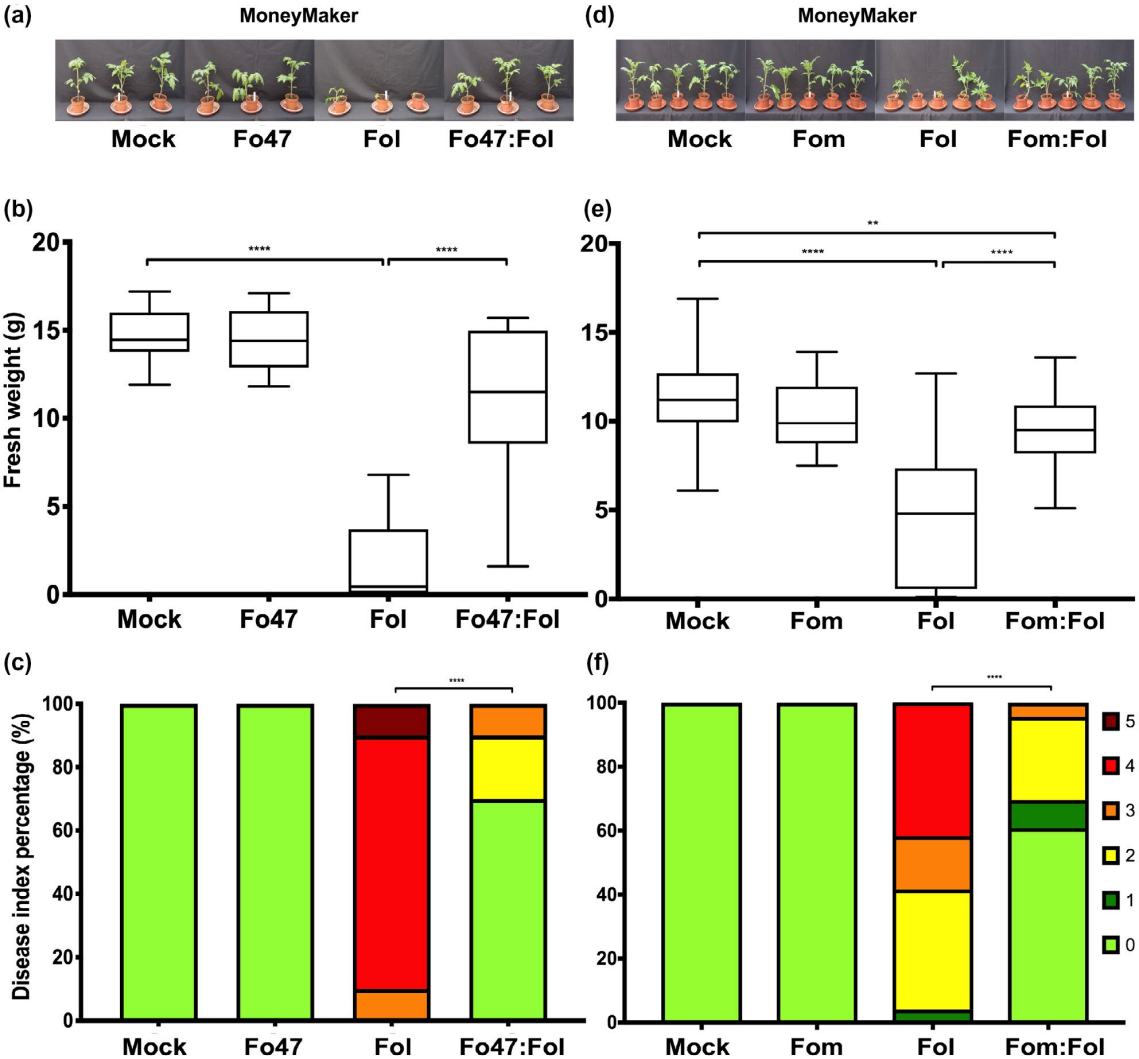
Both Fo47 and the melon pathogen*Fusarium oxysporum*(Fo) f. sp.*melonis*(Fom001) confer endophyte‐mediated resistance (EMR) to Fo f.sp.*lycopersici*(Fol4287) in tomato. (a) Ten‐day‐old MoneyMaker seedlings were root dip‐inoculated with water (mock), Fo47, Fom001, Fol, a mixture of Fo47 and Fol4287 (Fo47:Fol), or Fom001:Fol. In this figure a minimum of 10 seedlings was used for Fo47 treatment while 25 seedlings were used for the Fom treatments. (b) Three weeks postinoculation fresh weight (FW) and (c) disease index (DI) were scored. The experiment was repeated three times with 10, 15, or 25 plants/treatment showing similar results. (d), (e), and (f) The same bioassays were carried out with Fom001. An unpaired comparison for FW and DI was performed using the nonparametric Mann–Whitney*U*test (*p < .05, **p < .01, ****p < .0001)

Besides Fo endophytes such as Fo47, nonhost Fo pathogenic strains are also reported to confer EMR (Biles & Martyn, 1989; Díaz et al., 2005). Nonetheless, it is unknown whether nonhost Fo pathogens confer EMR in tomato to an extent similar to endophytic strains. To address this question, the EMR‐inducing potential of a melon‐pathogenic strain, Fom001, was assessed in tomato and compared to that of Fo47. The latter is a well‐known EMR inducer when coinoculated with Fol (Constantin et al., 2019; de Lamo et al., 2018). Ten‐day‐old MoneyMaker (MM) seedlings were root dip‐inoculated with water (mock), Fo47, the tomato pathogen Fol4287 or Fo47:Fol, a mixture of Fo47 and Fol4287 (Figure 1a). Three weeks postinoculation (wpi) Fo47 was found to confer EMR as fresh weight (FW) of plants coinoculated with Fo47 and Fol4287 was significantly higher than that of plants infected with Fol4287 alone (Figure 1b). In addition, the disease index (DI) was reduced when the pathogenic and Fo47 biocontrol strains were coinoculated as compared to infection solely with the pathogenic strain (Figure 1c). Using the same procedure Fom001 was found to confer EMR to Fol4287 to an extent similar to Fo47 (Figure 1d) as FW and DI were affected to a comparable extent in this tripartite interaction (Figure 1e,f). These findings show that the nonhost pathogen Fom001 is an endophyte of tomato and confers EMR, reducing disease symptoms caused by a tomato pathogenic Fo strain.

### 2.2 Avr2 compromises tomato responsiveness to a fungal MAMP

A tomato PTI‐mutant is instrumental to assess involvement of the PTI response in EMR. However, because PTI knockout mutants have not been described in tomato, we investigated whether the transgenic line MM‐∆*spAvr2‐*30 could be used as a proxy. This transgenic line accumulates Avr2 intracellularly, resulting in a compromised PTI response as exemplified by its reduced responsiveness to the bacterial MAMP flg22 (Di et al., 2016, 2017). If Avr2 targets a general component of PTI signalling, and not a flg22‐specific factor, then this line provides an excellent proxy of a PTI mutant to study the role of PTI in EMR in tomato.

**FIGURE 2:**
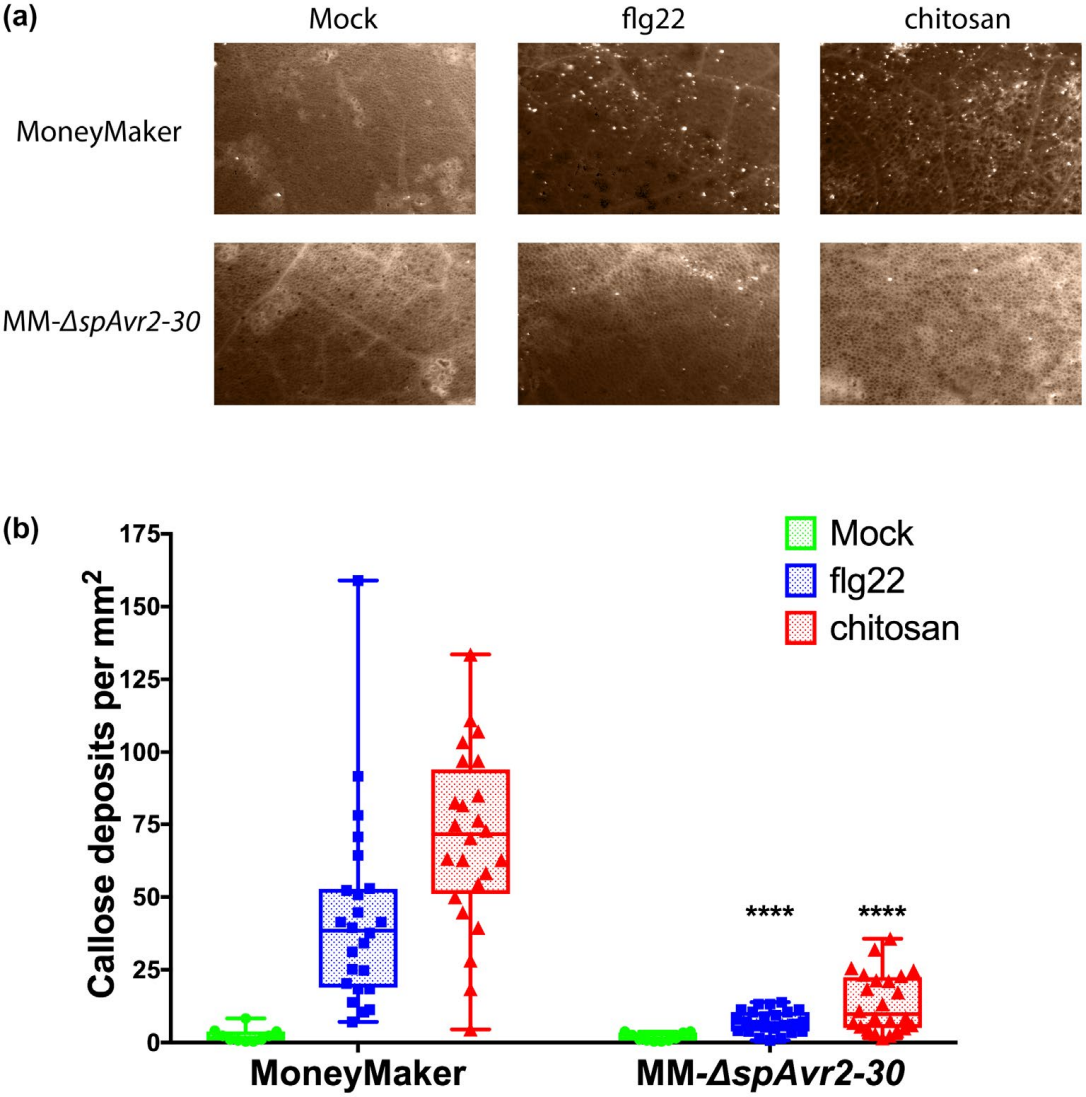
Compared to wild‐type MoneyMaker (MM) tomato, MM*‐*∆*spAvr2‐30*plants show a reduced number of callose depositions in leaves upon flg22 and chitosan infiltration. (a) flg22 (100 nM)‐ and chitosan (100 µg/ml)‐induced callose depositions were visualized by aniline blue staining in MM and MM*‐*∆*spAvr2‐30*leaves. (b) Quantification of flg22‐ and chitosan‐ induced callose deposits per mm^2^. Three MM plants and three MM*‐*∆*spAvr2‐30*plants were selected for flg22 or chitosan infiltration. Four leaf discs were taken per plant. For the mock treatments two plants were infiltrated. Three pictures were taken from different areas of the treated leaf discs. The experiment was replicated twice with the same results. An unpaired comparison was performed using the nonparametric Mann–Whitney*U*test (****p < .0001)

To test whether Avr2 affects the responsiveness of tomato to fungal MAMPs, leaves of MM and MM‐∆*spAvr2‐30* were infiltrated with the fungal MAMP‐derivative chitosan and subsequently callose depositions were monitored. Chitosan was used instead of chitin (poly‐*N*‐acetylglucosamine) as this deacetylated variant of chitin is more soluble and has been reported to induce a potent defence response in tomato (Rendina et al., 2019). As a positive control for PTI induction flg22 was used while Milli‐Q water served as negative control. Formation of callose deposits was detected in chitosan‐ and flg22‐treated leaves to a significantly higher extent than in water‐infiltrated leaves (Figure 2). This result shows that these depositions are a specific response to the MAMPs and are not due to the infiltration procedure. As expected, MM‐∆*spAvr2‐30* leaves infiltrated with flg22 showed significantly fewer callose depositions as compared to wild‐type MM leaves, confirming previous reports (Figure 2b; Di et al., 2017). Compared to wild‐type MM, MM‐∆*spAvr2‐30* leaves infiltrated with chitosan showed significantly fewer callose depositions, indicating that Avr2 also suppresses chitosan‐induced defence responses.

As mentioned, chitosan is a deacetylated form of chitin and this modification could affect receptor‐specific perception by the plant. To assess whether the chitosan preparation used in our studies triggers a receptor‐specific PTI response, *Arabidopsis* Col‐0 wild‐type and the *cerk1‐2* mutant were treated with chitosan and the formation of callose deposits was monitored. We observed that callose depositions were only induced in wild‐type Col‐1 plants, but not in the *cerk1‐2* mutant, indicating that the preparation contains a specific MAMP recognized by the CERK1 receptor (Figure S1a). As a positive control for PTI‐inducibility flg22 was used, and found to trigger a callose deposition response in both wild‐type and *cerk1‐2* mutant plants (Figure S1b). To test whether Avr2 targets a conserved component in chitosan‐ and flg22‐induced PTI signalling the callose deposition response in ∆*spAvr2* transgenic *Arabidopsis* was analysed (Figure S1c). Compared to the wild‐type Col‐0 plants a significantly reduced callose deposition was observed in the ∆*spAvr2* transgenic *Arabidopsis* upon chitosan and flg22 treatment (Figure S1). The observations that flg22‐ and chitosan‐induced PTI responses are compromised in both MM‐∆*spAvr2‐30* tomato and in ∆*spAvr2 Arabidopsis* indicate that the Avr2 effector targets a conserved—and shared—signalling component downstream of the respective PRR receptors, making ∆*spAvr2* transgenic lines a good proxy for a PTI mutant.

### 2.3 Endophytic *F. oxysporum* strains hypercolonize PTI‐compromised tomato

**FIGURE 3:**
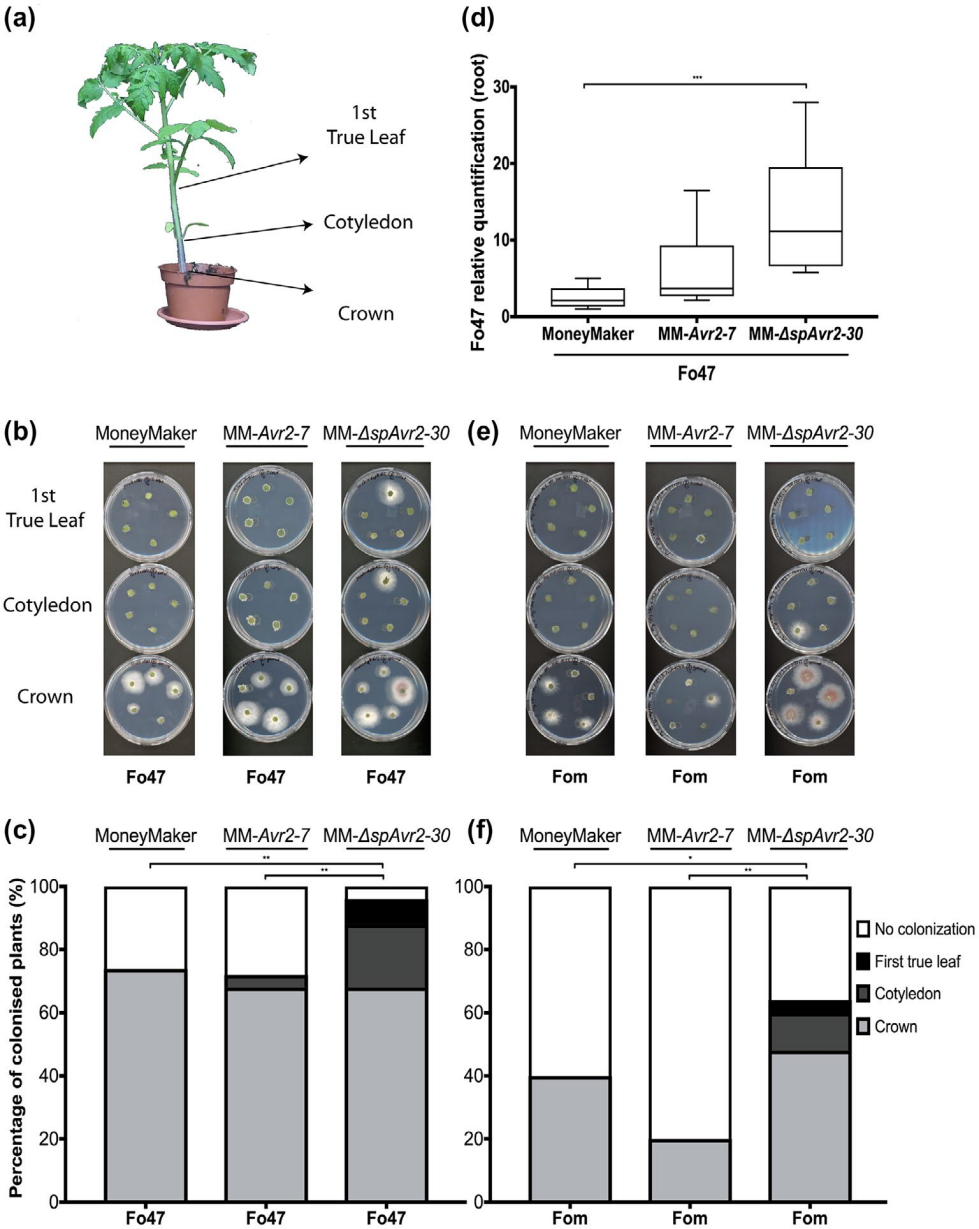
Intracellular presence of Avr2 in tomato facilitates*Fusarium oxysporum*(Fo) endophytism. (a) To monitor stem colonization by Fo endophytes stem sections from Fo47‐ or Fom001‐inoculated MoneyMaker, MM*‐Avr2‐7*, and MM*‐*∆*spAvr2‐30*were harvested at the crown, cotyledon, and first true leaf levels. Stems sections of Fo47 (hygromycin‐resistant)‐infected plants were surface‐sterilized and placed on (b) potato dextrose agar supplemented with hygromycin. Plates were scanned after 4 days of incubation at 25 °C in the darkness. In the pictures, a subset of five replicates out of a total of around 20 is shown. (c) Positive fungal outgrowth was quantified. The*y*axis represents the percentage of biological replicates that were (non‐)colonized to the crown, cotyledon, or first true leaf level. One representative bioassay is shown as illustration. (d) Root colonization by Fo47 was quantified by means of quantitative PCR. Three technical replicates were done for each biological replicate. (e) and (f) Stem colonization of Fom001 in the same tomato lines was also monitored. The experiments were replicated twice. An unpaired comparison for the relative quantification and the fungal stem reisolations was performed using the nonparametric Mann–Whitney *U* test (*p < .05, **p < .01, ***p < .001, ****p < .0001)

To establish themselves within the roots, endophytes are hypothesized to suppress and/or avoid activation of extensive host defences (Teixeira et al., 2019). To investigate whether PTI affects colonization by endophytic Fo of tomato, stem pieces from MM, MM‐*Avr2‐7*, and MM‐∆*spAvr2‐30* tomato plants were harvested 3 wpi with Fo47 (10, 15, or 25 plants/tomato genotype). Stem sections where taken at crown, cotyledon, and the first true leaf to monitor the extent of host colonization by the fungus (Figure 3a). Line MM‐*Avr2‐7* was included as a negative control in which PTI is not suppressed. In this line Avr2 is secreted into the apoplast while its virulence function has been reported to be intracellular (Di et al., 2017). Fungal outgrowth of the stem segments was scored after 4 days of incubation on agar plates (Figure 3b). As reported (Constantin et al., 2019; de Lamo et al., 2018), Fo47 is a poor stem colonizer as colonization of wild‐type MM was typically confined to the crown level (Figure 3c). Like wild‐type MM, MM‐*Avr2‐7* control plants were colonized only until the crown, with the exception of a single plant in which the fungus was observed at the cotyledon level (Figure 3c). Interestingly, in the PTI‐compromised MM‐∆*spAvr2‐30* line, Fo47 often reached the cotyledons and occasionally even the first true leaf (Figure 3c). Altogether, an effective PTI response to Fo47 appears to be instrumental in controlling the extent of host colonization by a Fo endophyte as MM‐∆*spAvr2‐30* plants are hypercolonized, as compared to wild‐type and MM‐*Avr2‐7* plants.

Next, we set out to quantitatively assess whether Fo47 colonization of stems correlated with a more extensive colonization of roots. Thereto genomic DNA was isolated from roots of inoculated wild‐type, MM‐*Avr2‐7*, and MM‐∆*spAvr2‐30* plants. Subsequently, quantitative PCR (qPCR) was performed with a Fo47‐specific primer pair to quantify fungal biomass relative to tomato *β‐tubulin*, which served as endogenous control (Figure 3d). Fo47‐inoculated MM wild‐type and MM‐*Avr2‐7* plants consistently showed the least colonization, while MM‐∆*spAvr2‐30* showed a significantly higher amount of fungal biomass in the three tomato lines analysed. These data show that a compromised PTI results in a more abundant colonization of tomato roots by the endophyte.

To assess whether PTI also restricts colonization of a nonhost pathogen, the MM, MM‐*Avr2‐7*, and MM‐∆*spAvr2‐30* tomato lines were inoculated with Fom001. Like Fo47, Fom001 was found to colonize MM‐∆*spAvr2‐30* to a higher extent than wild‐type MM and MM‐*Avr2‐7* plants. Colonization of the latter two genotypes was highly similar (Figure 3e,f), resembling the pattern observed for Fo47. Together, these experiments show that both Fo47 and Fom001 become hypercolonizers of PTI‐compromised tomato plants.

### 2.4 Endophyte‐mediated resistance is unaffected in PTI‐compromised tomato

**FIGURE 4:**
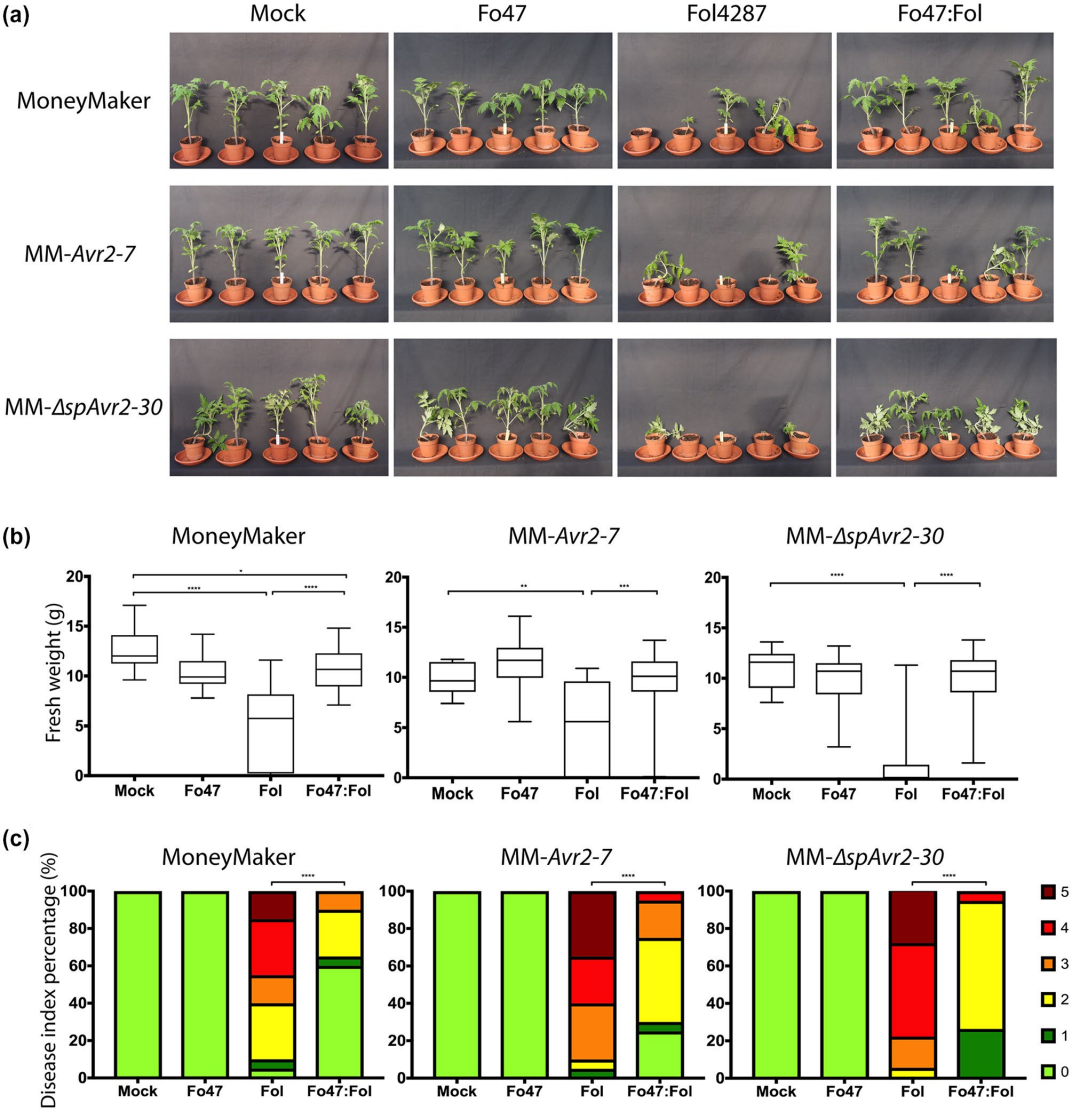
Fo47 does not lose its endophyte‐mediated resistance‐inducing capabilities in pattern‐triggered immunity‐compromised plants. (a) Ten‐day‐old MoneyMaker, MM*‐Avr2‐7*, and MM*‐*∆*spAvr2‐30*seedlings were root dip‐inoculated with water (mock), Fo47, Fol, or a mixture of Fo47:Fol4287. A minimum of 10 seedlings was used for the mock treatment and 20 were used for the other treatments. Three weeks postinoculation (b) fresh weight (FW) and (c) disease index (DI) were scored. The experiment was repeated three times with similar results. An unpaired comparison for FW and DI was performed using the nonparametric Mann–Whitney U test (*p < .05, **p< .01, ***p < .001, ****p < .0001)

**FIGURE 5:**
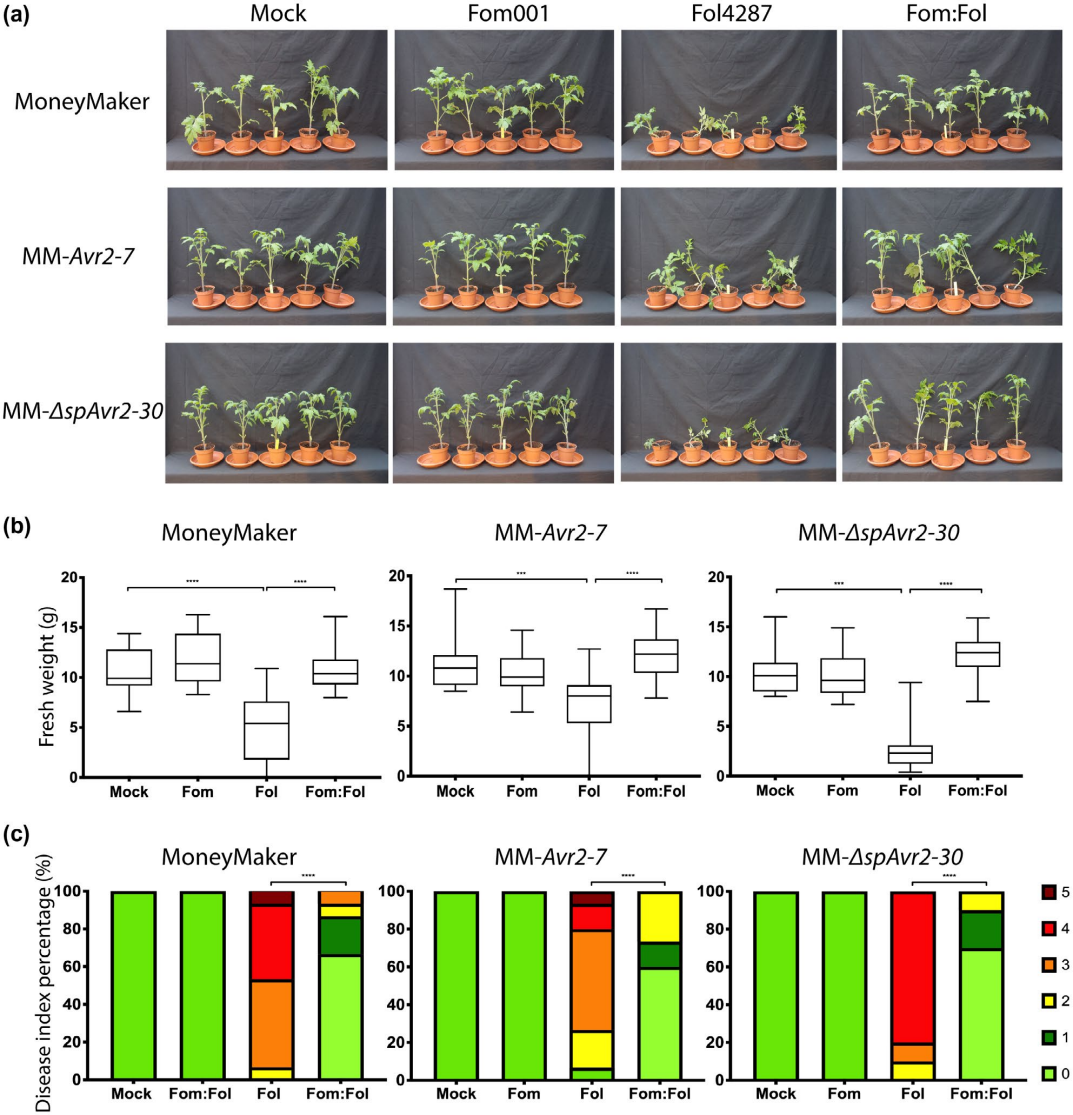
Melon pathogen Fom001 does not lose its endophyte‐mediated resistance‐inducing capabilities in pattern‐triggered immunity‐compromised plants. (a) Ten‐day‐old MoneyMaker, MM*‐Avr2‐7*, and MM*‐*∆*spAvr2‐30*seedlings were root dip‐inoculated with water (mock), Fom001, Fol,or a mixture of Fom001:Fol4287. Ten MM*‐*∆*spAvr2‐30*and 15 MM or MM*‐Avr2‐7*plants were used per treatment. Three weeks postinoculation (b) fresh weight (FW) and (c) disease index (DI) were scored. The experiment was repeated three times. An unpaired comparison for FW and DI was performed using the nonparametric Mann–Whitney*U*test (**p < .01, ****p < .0001)

Previous studies showed that endophytes can reduce host susceptibility to different pathogens (Ghorbanpour et al., 2018; de Lamo & Takken, 2020). We were interested in testing whether PTI affects EMR conferred by either Fo47 (Figure 4) or the melon pathogen Fom001 (Figure 5). MM, MM‐*Avr2‐7*, and MM‐∆*spAvr2‐30* tomato plants were inoculated with water (mock), Fo47, Fol4287, or a 1:1 mixture of both (Figure 4a). Fo47 was found to confer EMR to Fol4287 in the three different tomato genotypes as FW of coinoculated plants was always significantly higher than that of solely Fol4287‐infected plants (Figure 4b). In line with this observation, coinoculated plants showed fewer disease symptoms than solely Fol4287‐inoculated ones (Figure 4c). A similar result was found when MM, MM‐*Avr2‐7*, and MM‐∆*spAvr2‐30* were inoculated with Fom001 as EMR‐inducing strain (Figure 5a). Fom001 conferred EMR in all three tomato lines as FW was consistently higher in the coinoculated plants as compared to the plants inoculated with Fol4287 alone (Figure 5b). In one replicate inoculation of MM‐∆*spAvr2‐30* with Fom001 caused a reduction in the FW of the plants as compared to the mock. Fom001 was found not to cause any disease symptoms in any of the plant lines (Figure 5c). Altogether, EMR appears not to rely on PTI as the endophyte‐induced immune response was still observed in PTI‐compromised tomato.

## 3 DISCUSSION

We report that the endophyte Fo47 and the melon pathogen Fom001 become hypercolonizers of PTI‐compromised tomato plants. Surprisingly, the biocontrol conferred by both Fo strains towards Fol4287 was unaffected in these mutant plants. Our findings imply that EMR operates independently from PTI signalling. As proxy for a PTI mutant we used the transgenic MM‐∆*spAvr2‐30* line that produces the fungal effector protein Avr2, compromising PTI signalling. Previously, these plants have been reported to be hypersusceptible to various pathogens, including bacterial and fungal pathogens, and to exert a compromised immune response on treatment with a bacterial MAMP (Di et al., 2017). In this study we extend these findings by reporting that ∆*spAvr2* tomato, but also ∆*spAvr2 Arabidopsis* plants, are also compromised in their response to the fungal MAMP chitosan.

Hypercolonization of stems and roots of ∆*spAvr2* plants by Fom001 and Fo47 shows that PTI controls the extent of host colonization by nonpathogens. Hence, the hypothesized suppression and/or avoidance of activation of host defences by endophytes to colonize roots (Teixeira et al., 2019) does not seem to involve a complete suppression of PTI. Indeed, Fo47 infection has been reported to transiently induce immune signalling in tomato roots, resulting in papilla formation compromising host cell penetration by Fo47 (Benhamou et al., 2002). Our observation that PTI controls root colonization of Fo47 is interesting in the light of the recent finding that tissue damage is required for differentiated *Arabidopsis* roots to mount a full PTI response (Zhou et al., 2020). Spontaneous cell death, or cell death induced by the formation of lateral roots, nematode feeding, or pathogen presence, triggers PRR expression in neighbouring cells, enabling these cells to perceive MAMPs and mount a PTI response. Whether Fo‐induced host cell death is required for tomato roots to mount PTI remains a question for future study. The observation that Fo47 induces cell death near infection sites (Alabouvette et al., 2009), while pathogenic strains do not (Alabouvette et al., 2009; He et al., 2002; Humbert et al., 2015; Olivain et al., 2003), would be consistent with our finding that PTI is involved in restricting root colonization by the endophyte. Of note, although both Fom001 and Fo47 become hypercolonizers of PTI suppressed plants, these strains did not become pathogenic and did not cause disease symptoms. A possible explanation for this is that they lack the specific effector repertoire of Fol that is located on its pathogenicity chromosome. Transfer of this specific chromosome to Fo47 converts it into a genuine tomato pathogen, showing that this chromosome is required and sufficient for pathogenicity (Ma et al., 2010). Apparently, these Fol effectors have functions besides suppressing PTI that are required for pathogenicity and to cause disease on tomato.

The observation that EMR is still induced in ∆*spAvr2* plants can be explained by different nonexclusive models. One model identifies a plant‐directed response as the main factor in disease reduction, while the other proposes antagonism between the fungal strains as the main contributor. The latter scenario fits the interaction between the colocalizing fungi *Serendipita indica* and the root pathogen *Bipolaris sorokiniana* in barley roots in which the endophyte suppresses virulence of the latter by affecting expression of its effector genes and genes involved in secondary metabolism (Sarkar et al., 2019). In our system endophyte and pathogen colonize different plant compartments, root cortex versus vasculature, indicating an important contribution of a plant directed response. Especially during the later stages of infection there is a clear physical separation between the fungi as the pathogen colonizes the vasculature of the stems while the endophyte remains root‐confined (de Lamo et al., 2018). Furthermore, Fo47 and Fol do not appear to compete for infection sites on the root. When both fungi are coinoculated they colonize the same spots on the tomato root surfaces and cortex, and even when excess Fo47 spore concentrations are used Fol is still able to colonize these root niches and infect the host, indicative of a marginal role of niche competition as the main component of EMR (Olivain et al., 2006). In addition, as relatively low doses of Fo47 inoculum confer protection to Fol‐induced disease a plant‐mediated response is likely to be involved (Fravel et al., 2003). In our study a 1:1 ratio of endophyte and pathogen was used, but equally strong protection was found using a 1:10 ratio and even at a 1:100 ratio disease development was suppressed by the endophyte (Constantin et al., 2020). So, although direct competition between the strains might contribute to EMR, the host will certainly contribute to the observed resistance in this tripartite system.

Whereas our data strongly suggest that PTI is not involved in EMR, its involvement cannot completely be excluded as PTI is compromised but not fully absent in the MM‐∆*spAvr2‐30* and the ∆*spAvr2 Arabidopsis* lines. Chitosan‐induced callose depositions are, for instance, not completely absent in ∆*spAvr2* plants (Figures 2 and S1) as is phosphorylation of MAPKs upon flg22 treatment (Di et al., 2017). Completely excluding potential involvement of PTI in EMR awaits generation of stable tomato knockout mutants in which PTI signalling is practically absent, like the *bbc Arabidopsis* mutants (Xin et al., 2016). However, other lines of evidence also imply that PTI is not instrumental for EMR induction. PTI induction typically results in induction of expression of defence genes such as *PR‐1* and the encoded protein is not found in xylem sap of tomato plants coinoculated with Fo47 and Fol (de Lamo et al., 2018). The absence of this PTI marker suggests that either PTI is not, or is only transiently, induced during EMR. Furthermore, expression of SA marker genes (the SA biosynthesis genes *isochorismate synthase* [*ICS*] and *phenylalanine ammonia‐lyase* [*PAL*], or the *pathogenesis‐related 1a* [*PR1a*]), a JA reporter gene (*proteinase inhibitor* [*PI‐I*]), or ET‐regulated marker genes (*ethylene responsive factor* [*Pti4*] and *ethylene receptor* [*ETR4*]) are unaffected by EMR (Constantin et al., 2019). Together with the observation that EMR is still induced in tomato lines that are compromised in either JA, ET, or SA signalling (Constantin et al., 2019), these data provide an argument for EMR being distinct from the known induced resistance responses that rely on these hormones and are triggered by PTI (Mishina & Zeier, 2007).

Taken together, our data support a hypothesis in which Fo‐induced EMR represents a type of induced resistance that is distinct from the other induced resistance responses in plants, such as SAR and ISR, as it is still functional in tomato plants in which PTI signalling is compromised. This response seems specifically potent to control wilt disease caused by pathogenic Fo strains and to root‐invading pathogens (de Lamo & Takken, 2020). A detailed molecular understanding of the mechanisms underlying in Fo‐based EMR will be instrumental in developing novel biocontrol strategies to limit our dependency on pesticides in agriculture.

## 4 EXPERIMENTAL PROCEDURES

### 4.1 Plant and fungal materials and cultivation conditions

Three different tomato lines were used that are susceptible to Fol4287 (FP3059): wild‐type MoneyMaker (MM), a MM‐*Avr2‐7* line constitutively expressing the Fol *Avr2* effector gene, and a MM*‐*∆*spAvr2‐30* line constitutively producing a Fol Avr2 variant lacking its signal peptide resulting in a cytoplasmically localized Avr2 protein (Di et al., 2016). Besides wild‐type Col‐0, an ∆*spAvr2‐23* line (Di et al., 2016) and a *cerk1‐2* mutant (Miya et al., 2007) were used. Fungal infections were carried out with Fo strains carrying a gene that confers resistance to hygromycin. The endophytic strains used were Fo47 (FP1544) and Fom001 (FP1577); the latter is a pathogen on melon. As pathogenic strain, Fol4287 was used. Tomato plants were grown in a climate‐controlled greenhouse at 25 °C, 65% relative humidity and a 16 hr photoperiod. *Arabidopsis* plantlets were grown under short day conditions, 13 hr/11 hr dark/light cycles at 22 °C.

### 4.2 Chitosan and flg22 preparation

Chitosan with a low molecular weight (50–190 kDa, 75%–85% deacetylated; Sigma‐Aldrich) was prepared as described (Rendina et al., 2019). In brief, chitosan was dissolved in 0.2 M acetic acid to a concentration of 1 g/ml, stirred overnight, and diluted in Milli‐Q water to a concentration of 5 mg/ml. Flg22 was dissolved in sterile Milli‐Q water at a concentration of 100 μM.

### 4.3 Callose deposition assays and microscopy

Leaves of 3‐week‐old tomato plants or 4‐week‐old *Arabidopsis* plants were syringe‐infiltrated with water, flg22 (100 nM), or chitosan (100 μg/ml) and after 24 hr leaf discs were taken from the infiltrated areas. Leaf discs were stored in 70% ethanol: acetic acid (3:1). Cleared leaf discs were washed and rehydrated in 50% ethanol and Milli‐Q water. Staining was done for 60–120 min with a 0.01% aniline blue solution (dissolved in 0.07 M sodium phosphate buffer) at pH 9. The samples were mounted in 50% glycerol and callose was visualized using a Leica MZ FLIII fluorescence microscope with a DAPI filter (UV filter, excitation 360 nm, emission 420 nm). The number of callose depositions within a frame (5× or 8× magnification) was counted using Fiji (https://imagej.net/Fiji). A Mann–Whitney *U* statistical test was applied on the number of callose deposits in tomato and a one‐way analysis of variance (ANOVA) was used for the number of *Arabidopsis* deposits using the software PRISM 7.0 (GraphPad).

### 4.4 *Fusarium* inoculation assays

Hygromycin‐resistant Fo47, Fom001, or Fol4287 were inoculated from glycerol stock to potato dextrose agar (PDA) plates supplemented with 100 mg/L hygromycin. Plate cultures were grown for at least 5 days at 25 °C in darkness. Agar plugs were used to inoculate 100 ml minimal medium (0.17% yeast nitrogen base without amino acids or ammonium sulphate, 3% sucrose and 100 mM KNO_3_). Cultures were incubated in the dark at 25 °C and 150 rpm for 5 days. Thereafter, cultures were filtered through Miracloth (Calbiochem) and diluted to generate a microconidial inoculum of 10^7^ spores/ml (Di et al., 2016; de Lamo et al., 2018). Coinoculum of endophyte (Fo47 or Fom001) and pathogen (Fol4287) was prepared in a 1:1 ratio (10^7^ microconidia/ml each). Subsequently, 10‐day‐old tomato seedlings (MM, MM‐*Avr2‐7*, and MM‐∆*spAvr2‐30*) were uprooted and the roots were trimmed at 1 cm to promote fungal infection. Seedlings were root dip‐inoculated by placing them for 5 min in water (mock) or a microconidial suspension of the endophyte (Fo47 or Fom001), pathogen (Fol4287), or a mixture of both. Seedlings were repotted and 3 weeks postinoculation (wpi) fresh weight (FW) and disease index (DI) were scored as described (de Lamo et al., 2018). A Mann–Whitney *U* test was applied on the FW and DI using the software PRISM 7.0 (GraphPad).

### 4.5 Fungal stem reisolation assays

Three weeks after inoculation stem pieces were collected and analysed for fungal presence. Stem pieces were surface sterilized with 70% ethanol for approximately 3 min and subsequently rinsed with sterile water. From each stem piece, one slice (c.0.5 cm thick) was cut from the crown, cotyledon, and first true leaf segment (Figure 3a) and placed on PDA plates supplemented with penicillin (100 μg/ml) and streptomycin (200 μg/ml) to prevent bacterial growth. Additionally, the PDA plates contained hygromycin (100 μg/ml) to select for Fo. The plates were kept at 25 °C for 4 days and then scanned using an EPSON Perfection V800 Photo scanner and processed with the software package SilverFast 8 (LaserSoft Imaging). Thereafter fungal outgrowth was analysed. For statistical analyses, a 0 was given to noncolonized plants, 1 for plants that were colonized until the crown, 2 for colonization until the cotyledon and 3 for colonization until the first true leaf level. These categories were analysed for statistical differences using a Mann–Whitney *U* test.

### 4.6 Relative quantification of *Fusarium* root colonization by qPCR

Three wpi roots from Fo47‐inoculated MM, MM‐*Avr2‐7*, and MM‐∆*spAvr2‐30* plants were harvested and snap‐frozen into liquid nitrogen. Root tissue was thoroughly ground in a mortar and genomic DNA (gDNA) isolation was performed with the GeneJET Plant Genomic DNA Purification Mini Kit (Thermo Scientific). The isolated gDNA was used for real‐time quantitative PCR (qPCR). Each qPCR contained 1 μl of gDNA (c.50 ng), 3 μl Milli‐Q water, 2 μl of 5 × HOT FIREPol EvaGreen qPCR Mix Plus (ROX) (Solis BioDyne), 2 μl of 5 pmol/μl forward primer (PF), and 2 μl of 5 pmol/μl reverse primer (PR). Tomato *β*‐*tubulin* was used as an endogenous reference gene for normalizing fungal gDNA to tomato gDNA (*β‐tubulin* primers PF: 5′‐CAGTGAAACTGGAGCTGGAA‐3′; PR: 5′‐TATAGTGGCCACGAGCAAAG‐3′). A primer pair targeting a Fo47‐specific SCAR region was used to amplify fungal DNA (PF: 5′‐CCTCAACTTCTGATTTAAATATGA‐3′; PR: 5′‐GAGCGAACAACTACAATAAAAG‐3′) (Edel‐Hermann et al., 2011). PCRs were done in 96‐well plates, using a QuantStudio 3 thermocycler (Applied Biosystems). The qPCR programme started with 95 °C for 15 min; followed by 40 cycles of 95 °C for 15 s, 58 °C for 20 s, and 60 °C for 30 s; and a final step to generate a melting curve that consisted of 95 °C for 15 s, 60 °C for 1 min, and 95 °C for 15 s. Data analysis was performed using the webtool provided by Thermo Scientific (https://apps.thermofisher.com). A Mann–Whitney *U* test was applied using the software PRISM 7.0 (GraphPad).

## CONFLICT OF INTEREST

The authors declare no conflict of interest.

## Supporting information


**FIGURE S1** Compared to wild‐type *Arabidopsis*, *∆spAvr2‐25* plants show a reduced number of callose depositions in leaves upon chitosan and flg22 infiltration. (a) flg22 (100 nM)‐ and chitosan (100 µg/ml)‐induced callose depositions were visualized by aniline blue staining in Col‐0, *cerk1‐2*, and *∆spAvr2‐25* leaves. (b) Quantification of flg22‐ and (c) chitosan‐induced callose deposits per frame. Three Col‐0, *cerk1‐2*, and *spAvr2‐25* plants were selected for mock, flg22, or chitosan infiltration. Four leaves were infiltrated per plant. Depending on the size of the leaves, two to four pictures were taken from different areas of the treated leaves. The experiment was replicated three times with similar results. An unpaired comparison was performed using one‐way analysis of variance (*p < .05, **p < .01, ***p < .001, ****p < .0001)

## Data Availability

The data that support the findings of this study are available from the corresponding author upon reasonable request.

## References

[mpp13018-bib-0001] Agrios, G.N. (2005) Plant Pathology 5th edition. Burlington, MA: Elsevier Academic Press.

[mpp13018-bib-0002] Aïcha, B. , Nisserine, H.K. , Abdeslem, S.M. , Hadjira, B. , Lalia, B.S. , Mebrouk, K. et al. (2014) Antagonist effect of nonpathogenic *Fusarium oxysporum* strain *Fo*47 on *Fusarium oxysporum* f. sp. *radicis‐lycopersici* the cause of fusarium crown and root rot in tomato cultivation. Advances in Environmental Biology, 8, 50–56.

[mpp13018-bib-0003] Aimé, S. , Alabouvette, C. , Steinberg, C. & Olivain, C. (2013) The endophytic strain *Fusarium oxysporum* Fo47: a good candidate for priming the defense responses in tomato roots. Molecular Plant‐Microbe Interactions, 26, 918–926. 23617416 10.1094/MPMI-12-12-0290-R

[mpp13018-bib-0004] Alabouvette, C. , Olivain, C. , Migheli, Q. & Steinberg, C. (2009) Microbiological control of soil‐borne phytopathogenic fungi with special emphasis on wilt‐inducing *Fusarium oxysporum* . New Phytologist, 184, 529–544. 10.1111/j.1469-8137.2009.03014.x 19761494

[mpp13018-bib-0005] Benhamou, N. , Garand, C. & Goulet, A. (2002) Ability of nonpathogenic *Fusarium oxysporum* strain Fo47 to induce resistance against *Pythium ultimum* infection in cucumber. Applied and Environmental Microbiology, 68, 4044–4060. 12147506 10.1128/AEM.68.8.4044-4060.2002 PMC124014

[mpp13018-bib-0006] Bigeard, J. , Colcombet, J. & Hirt, H. (2015) Signaling mechanisms in pattern‐triggered immunity (PTI). Molecular Plant, 8, 521–539. 25744358 10.1016/j.molp.2014.12.022

[mpp13018-bib-0007] Biju, V.C. , Fokkens, L. , Houterman, P.M. , Rep, M. & Cornelissen, B.J.C. (2017) Multiple evolutionary trajectories have led to the emergence of races in *Fusarium oxysporum* f. sp *lycopersici* . Applied and Environmental Microbiology, 83, e02548–e2616. 27913420 10.1128/AEM.02548-16 PMC5288826

[mpp13018-bib-0008] Biles, C.L. & Martyn, R.D. (1989) Local and systemic resistance induced in watermelons by formae speciales of *Fusarium oxysporum* . Phytopathology, 79, 856–860.

[mpp13018-bib-0009] Bolwerk, A. , Lagopodi, A.L. , Lugtenberg, B.J.J. & Bloemberg, G.V. (2005) Visualization of interactions between a pathogenic and a beneficial *Fusarium* strain during biocontrol of tomato foot and root rot. Molecular Plant‐Microbe Interactions, 18, 710–721. 16042017 10.1094/MPMI-18-0710

[mpp13018-bib-0010] Catanzariti, A.M. , Do, H.T.T. , Bru, P. , de Sain, M. , Thatcher, L.F. , Rep, M. et al. (2017). The tomato *I* gene for fusarium wilt resistance encodes an atypical leucine‐rich repeat receptor‐like protein whose function is nevertheless dependent on SOBIR1 and SERK3/BAK1. The Plant Journal, 89, 1195–1209. 27995670 10.1111/tpj.13458

[mpp13018-bib-0011] Catanzariti, A.M. , Lim, G.T.T. & Jones, D.A. (2015) The tomato*I‐3* gene: a novel gene for resistance to fusarium wilt disease. New Phytologist, 207, 106–118. 10.1111/nph.13348 25740416

[mpp13018-bib-0012] Chuberre, C. , Plancot, B. , Driouich, A. , Moore, J.P. , Bardor, M. , Gugi, B. et al. (2018) Plant immunity is compartmentalized and specialized in roots. Frontiers in Plant Science, 9, 1692. 30546372 10.3389/fpls.2018.01692 PMC6279857

[mpp13018-bib-0013] Constantin, M.E. , de Lamo, F.J. , Vlieger, B.V. , Rep, M. & Takken, F.L.W. (2019) Endophyte‐mediated resistance in tomato to *Fusarium oxysporum* is independent of ET, JA, and SA. Frontiers in Plant Science, 10, 979. 31417594 10.3389/fpls.2019.00979 PMC6685397

[mpp13018-bib-0014] Constantin, M.E. , Vlieger, B.V. , Takken, F.L.W. & Rep, M. (2020) Diminished pathogen and enhanced endophyte colonization upon coinoculation of endophytic and pathogenic *Fusarium* strains. Microorganisms, 8, 544. 10.3390/microorganisms8040544 PMC7232452 32283705

[mpp13018-bib-0015] Di, X. , Cao, L. , Hughes, R.K. , Tintor, N. , Banfield, M.J. & Takken, F.L.W. (2017) Structure–function analysis of the *Fusarium oxysporum* Avr2 effector allows uncoupling of its immune‐suppressing activity from recognition. New Phytologist, 216, 897–914. 10.1111/nph.14733 PMC5659127 28857169

[mpp13018-bib-0016] Di, X. , Gomila, J. , Ma, L. , van den Burg, H.A. & Takken, F.L.W. (2016) Uptake of the *Fusarium* effector Avr2 by tomato is not a cell autonomous event. Frontiers in Plant Science, 7, 1915. 28066471 10.3389/fpls.2016.01915 PMC5175262

[mpp13018-bib-0017] Díaz, J. , Silvar, C. , Varela, M.M. , Bernal, A. & Merino, F. (2005) *Fusarium* confers protection against several mycelial pathogens of pepper plants. Plant Pathology, 54, 773–780.

[mpp13018-bib-0018] Edel‐Hermann, V. , Aimé, S. , Cordier, C. , Olivain, C. , Steinberg, C. & Alabouvette, C. (2011) Development of a strain‐specific real‐time PCR assay for the detection and quantification of the biological control agent Fo47 in root tissues. FEMS Microbiology Letters, 322, 34–40. 21658107 10.1111/j.1574-6968.2011.02332.x

[mpp13018-bib-0019] Edel‐Hermann, V. & Lecomte, C. (2019) Current status of *Fusarium oxysporum formae speciales* and races. Phytopathology, 109, 512–530. 30461350 10.1094/PHYTO-08-18-0320-RVW

[mpp13018-bib-0020] Elmer, W.H. (2004) Combining nonpathogenic strains of *Fusarium oxysporum* with sodium chloride to suppress fusarium crown rot of asparagus in replanted fields. Plant Pathology, 53, 751–758.

[mpp13018-bib-0021] Fradin, E.F. , Zhang, Z. , Ayala, J.C.J. , Castroverde, C.D.M. , Nazar, R.N. , Robb, J. et al. (2009) Genetic dissection of *Verticillium* wilt resistance mediated by tomato *Ve1* . Plant Physiology, 150, 320–332. 19321708 10.1104/pp.109.136762 PMC2675724

[mpp13018-bib-0022] Fravel, D. , Olivain, C. & Alabouvette, C. (2003) *Fusarium oxysporum* and its biocontrol. New Phytologist, 157, 493–502. 10.1046/j.1469-8137.2003.00700.x 33873407

[mpp13018-bib-0023] Fu, Z.Q. & Dong, X.N. (2013) Systemic acquired resistance: turning local infection into global defense. Annual Review of Plant Biology, 64, 839–863. 10.1146/annurev-arplant-042811-105606 23373699

[mpp13018-bib-0024] Ghorbanpour, M. , Omidvari, M. , Abbaszadeh‐Dahaji, P. , Omidvar, R. & Kariman, K. (2018) Mechanisms underlying the protective effects of beneficial fungi against plant diseases. Biological Control, 117, 147–157.

[mpp13018-bib-0025] Gonzalez‐Cendales, Y. , Catanzariti, A.M. , Baker, B. , McGrath, D.J. & Jones, D.A. (2016) Identification of *I‐7* expands the repertoire of genes for resistance to fusarium wilt in tomato to three resistance gene classes. Molecular Plant Pathology, 17, 448–463. 26177154 10.1111/mpp.12294 PMC6638478

[mpp13018-bib-0026] Gordon, T.R. (2017) *Fusarium oxysporum* and the fusarium wilt syndrome. Annual Review of Phytopathology, 55, 23–39. 10.1146/annurev-phyto-080615-095919 28489498

[mpp13018-bib-0027] Hacquard, S. , Spaepen, S. , Garrido‐Oter, R. & Schulze‐Lefert, P. (2017) Interplay between innate immunity and the plant microbiota. Annual Review of Phytopathology, 55, 565–589. 10.1146/annurev-phyto-080516-035623 28645232

[mpp13018-bib-0028] He, C.Y. , Hsiang, T. & Wolyn, D.J. (2002) Induction of systemic disease resistance and pathogen defence responses in *Asparagus officinalis* inoculated with nonpathogenic strains of *Fusarium oxysporum* . Plant Pathology, 51, 225–230.

[mpp13018-bib-0029] Houterman, P.M. , Cornelissen, B.J. & Rep, M. (2008) Suppression of plant resistance gene‐based immunity by a fungal effector. PLoS Pathogens, 4, e1000061. 18464895 10.1371/journal.ppat.1000061 PMC2330162

[mpp13018-bib-0052] Houterman P. M. , Ma L. , van Ooijen G. , de Vroomen M. J. , Cornelissen B. J. C. , Takken F. L. W. , Rep M. (2009). The effector protein Avr2 of the xylem‐colonizing fungus *Fusarium oxysporum* activates the tomato resistance protein I‐2 intracellularly. The Plant Journal, 58, 970–978. 19228334 10.1111/j.1365-313X.2009.03838.x

[mpp13018-bib-0030] Humbert, C. , Aimé, S. , Alabouvette, C. , Steinberg, C. & Olivain, C. (2015) Remodelling of actin cytoskeleton in tomato cells in response to inoculation with a biocontrol strain of *Fusarium oxysporum* in comparison to a pathogenic strain. Plant Pathology, 64, 1366–1374.

[mpp13018-bib-0031] Jones, J.D.G. & Dangl, J.L. (2006) The plant immune system. Nature, 444, 323–329. 17108957 10.1038/nature05286

[mpp13018-bib-0032] de Lamo, F.J. , Constantin, M.E. , Fresno, D.H. , Boeren, S. , Rep, M. & Takken, F.L.W. (2018) Xylem sap proteomics reveals distinct differences between *R* gene‐ and endophyte‐mediated resistance against fusarium wilt disease in tomato. Frontiers in Microbiology, 9, 2977. 30564219 10.3389/fmicb.2018.02977 PMC6288350

[mpp13018-bib-0033] de Lamo, F.J. & Takken, F.L.W. (2020) Biocontrol by *Fusarium oxysporum* using endophyte‐mediated resistance. Frontiers in Plant Science, 11, 37. 32117376 10.3389/fpls.2020.00037 PMC7015898

[mpp13018-bib-0053] Ma L.‐J. , van der Does H. C. , Borkovich K. A. , Coleman J. J. , Daboussi M.‐J. , Di Pietro A. , et al. (2010) Comparative genomics reveals mobile pathogenicity chromosomes in *Fusarium* . Nature, 464, 367–373. 20237561 10.1038/nature08850 PMC3048781

[mpp13018-bib-0034] Macho, A.P. & Zipfel, C. (2014) Plant PRRs and the activation of innate immune signaling. Molecular Cell, 54, 263–272. 24766890 10.1016/j.molcel.2014.03.028

[mpp13018-bib-0035] Michielse, C.B. & Rep, M. (2009) Pathogen profile update: *Fusarium oxysporum* . Molecular Plant Pathology, 10, 311–324. 19400835 10.1111/j.1364-3703.2009.00538.x PMC6640313

[mpp13018-bib-0036] Mishina, T.E. & Zeier, J. (2007) Pathogen‐associated molecular pattern recognition rather than development of tissue necrosis contributes to bacterial induction of systemic acquired resistance in Arabidopsis. The Plant Journal, 50, 500–513. 17419843 10.1111/j.1365-313X.2007.03067.x

[mpp13018-bib-0037] Miya, A. , Albert, P. , Shinya, T. , Desaki, Y. , Ichimura, K. , Shirasu, K. et al. (2007) CERK1, a LysM receptor kinase, is essential for chitin elicitor signaling in Arabidopsis. Proceedings of the National Academy of Sciences of the United States of America, 104, 19613–19618. 18042724 10.1073/pnas.0705147104 PMC2148337

[mpp13018-bib-0054] Olivain C. , Humbert C. , Nahalkova J. , Fatehi J. , L'Haridon F. , Alabouvette C. (2006) Colonization of tomato root by pathogenic and nonpathogenic *Fusarium oxysporum* strains inoculated together and separately into the soil. Applied and Environmental Microbiology, 72, 1523–1531. 16461707 10.1128/AEM.72.2.1523-1531.2006 PMC1392888

[mpp13018-bib-0038] Olivain, C. , Trouvelot, S. , Binet, M.N. , Cordier, C. , Pugin, A. & Alabouvette, C. (2003) Colonization of flax roots and early physiological responses of flax cells inoculated with pathogenic and nonpathogenic strains of *Fusarium oxysporum* . Applied and Environmental Microbiology, 69, 5453–5462. 12957934 10.1128/AEM.69.9.5453-5462.2003 PMC194917

[mpp13018-bib-0039] Pieterse, C.M.J. , Zamioudis, C. , Berendsen, R.L. , Weller, D.M. , Van Wees, S.C.M. & Bakker, P.A.H.M. (2014) Induced systemic resistance by beneficial microbes. Annual Review of Phytopathology, 52, 347–375. 10.1146/annurev-phyto-082712-102340 24906124

[mpp13018-bib-0040] Postma, J. , Liebrand, T.W.H. , Bi, G.Z. , Evrard, A. , Bye, R.R. , Mbengue, M. et al. (2016) Avr4 promotes Cf‐4 receptor‐like protein association with the BAK1/SERK3 receptor‐like kinase to initiate receptor endocytosis and plant immunity. New Phytologist, 210, 627–642. 10.1111/nph.13802 26765243

[mpp13018-bib-0041] Rendina, N. , Nuzzaci, M. , Scopa, A. , Cuypers, A. & Sofo, A. (2019) Chitosan‐elicited defense responses in cucumber mosaic virus (CMV)‐infected tomato plants. Journal of Plant Physiology, 234, 9–17. 30640158 10.1016/j.jplph.2019.01.003

[mpp13018-bib-0042] Salerno, M.I. , Gianinazzi, S. & Gianinazzi‐Pearson, V. (2000) Effects on growth and comparison of root tissue colonization patterns of *Eucalyptus viminalis* by pathogenic and nonpathogenic strains of *Fusarium oxysporum* . New Phytologist, 146, 317–324. 10.1046/j.1469-8137.2000.00629.x 33862965

[mpp13018-bib-0043] Sarkar, D. , Rovenich, H. , Jeena, G. , Nizam, S. , Tissier, A. , Balcke, G.U. et al. (2019) The inconspicuous gatekeeper: endophytic *Serendipita vermifera* acts as extended plant protection barrier in the rhizosphere. New Phytologist, 224, 886–901. 10.1111/nph.15904 31074884

[mpp13018-bib-0044] Simons, G. , Groenendijk, J. , Wijbrandi, J. , Reijans, M. , Groenen, J. , Diergaarde, P. et al. (1998) Dissection of the *Fusarium I2* gene cluster in tomato reveals six homologs and one active gene copy. The Plant Cell, 10, 1055–1068. 9634592 10.1105/tpc.10.6.1055 PMC144031

[mpp13018-bib-0045] Takken, F.L.W. & Rep, M. (2010) The arms race between tomato and *Fusarium oxysporum* . Molecular Plant Pathology, 11, 309–314. 20447279 10.1111/j.1364-3703.2009.00605.x PMC6640361

[mpp13018-bib-0046] Teixeira, P.J.P. , Colaianni, N.R. , Fitzpatrick, C.R. & Dangl, J.L. (2019) Beyond pathogens: microbiota interactions with the plant immune system. Current Opinion in Microbiology, 49, 7–17. 31563068 10.1016/j.mib.2019.08.003

[mpp13018-bib-0047] Trouvelot, S. , Olivain, C. , Recorbet, G. , Migheli, Q. & Alabouvette, C. (2002) Recovery of *Fusarium oxysporum* Fo47 mutants affected in their biocontrol activity after transposition of the Fot1 element. Phytopathology, 92, 936–945. 18944018 10.1094/PHYTO.2002.92.9.936

[mpp13018-bib-0048] Veloso, J. & Díaz, J. (2012) *Fusarium oxysporum* Fo47 confers protection to pepper plants against *Verticillium dahliae* and *Phytophthora capsici*, and induces the expression of defence genes. Plant Pathology, 61, 281–288.

[mpp13018-bib-0049] Xin, X.F. , Nomura, K. , Aung, K. , Velasquez, A.C. , Yao, J. , Boutrot, F. et al. (2016) Bacteria establish an aqueous living space in plants crucial for virulence. Nature, 539, 524–529. 27882964 10.1038/nature20166 PMC5135018

[mpp13018-bib-0050] Yadeta, K.A. & Thomma, B.P.H.J. (2013) The xylem as battleground for plant hosts and vascular wilt pathogens. Frontiers in Plant Science, 4, 97. 23630534 10.3389/fpls.2013.00097 PMC3632776

[mpp13018-bib-0051] Zhou F. , Emonet A. , Dénervaud Tendon V. , Marhavy P. , Wu D. , Lahaye T. , Geldner N. (2020) Co‐incidence of damage and microbial patterns controls localized immune responses in roots. Cell, 180, 440–453.e18. 32032516 10.1016/j.cell.2020.01.013 PMC7042715

