# Supplementary figures and images for: Pattern‐triggered immunity restricts host colonization by endophytic fusaria, but does not affect endophyte‐mediated resistance
Source: Mol Plant Pathol. 2020 Nov 18;22(2):204–15. doi: 10.1111/mpp.13018 (PMC7814963; doi:10.1111/mpp.13018)

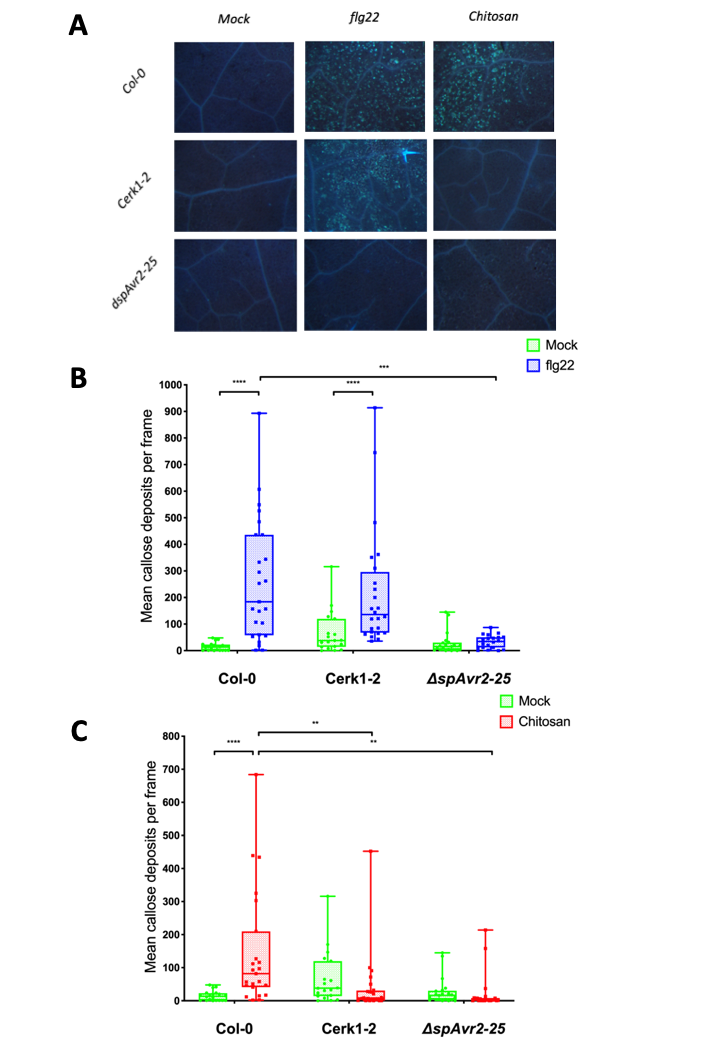

Supplement: Supplementary file 1 — FIGURE S1 Compared to wild‐type Arabidopsis, ∆spAvr2‐25 plants show a reduced number of callose depositions in leaves upon chitosan and flg22 infiltration. (a) flg22 (100 nM)‐ and chitosan (100 µg/ml)‐induced callose depositions were visualized by aniline blue staining in Col‐0, cerk1‐2, and ∆spAvr2‐25 leaves. (b) Quantification of flg22‐ and (c) chitosan‐induced callose deposits per frame. Three Col‐0, cerk1‐2, and spAvr2‐25 plants were selected for mock, flg22, or chitosan infiltration. Four leaves were infiltrated per plant. Depending on the size of the leaves, two to four pictures were taken from different areas of the treated leaves. The experiment was replicated three times with similar results. An unpaired comparison was performed using one‐way analysis of variance (*p < .05, **p < .01, ***p < .001, ****p < .0001) [file MPP-22-204-s001.tiff]
